# Optimization of the Selenization Temperature on the Mn-Substituted Cu_2_ZnSn(S,Se)_4_ Thin Films and Its Impact on the Performance of Solar Cells

**DOI:** 10.3390/nano12223994

**Published:** 2022-11-12

**Authors:** Zhanwu Wang, Yingrui Sui, Meiling Ma, Tianyue Wang

**Affiliations:** 1 Department of Life Sciences, Jilin Normal University, Siping 136000, China; 2 Key Laboratory of Functional Materials Physics and Chemistry of Ministry of Education, Jilin Normal University, Changchun 130103, China

**Keywords:** Cu_2_ZnSn(S,Se)_4_ film, Mn-doping, solar cells, properties, selenization temperature

## Abstract

Cu_2_ZnSn(S,Se)_4_ (CZTSSe) films are considered to be promising materials in the advancement of thin-film solar cells. In such films, the amounts of S and Se control the bandgap. Therefore, it is crucial to control the concentration of S/Se to improve efficiency. In this study, Cu_2_Mn_x_Zn_1−x_SnS_4_ (CMZTS) films were fabricated using the sol-gel method and treated in a Se environment. The films were post-annealed in a Se atmosphere at various temperature ranges from 300 °C to 550 °C at intervals of 200 °C for 15 min to obtain Cu_2_Mn_x_Zn_1−x_Sn(S,Se)_4_ (CMZTSSe). The elemental properties, surface morphology, and electro-optical properties of the CMZTSSe films were investigated in detail. The bandgap of the CMZTSSe films was adjustable in the scope of 1.11–1.22 eV. The structural propeties and phase purity of the CMZTSSe films were analyzed by X-ray diffraction and Raman analysis. High-quality CMZTSSe films with large grains could be acquired by suitably changing the selenization temperature. Under the optimized selenization conditions, the efficiency of the fabricated CMZTSSe device reached 3.08%.

## 1. Introduction

Cu_2_ZnSn(S,Se)_4_ (CZTSSe)-based solar cells are considered to constitute a promising candidate for future renewable energy materials. CZTSSe absorbers are made up of non-toxic and earth-abundant elements; they exhibit a high absorption coefficient of 10^4^ cm^−1^ and a bandgap energy (1.0–1.5 eV) that can be obtained by adjusting the ratio of S/Se [1,2,3]. Although its highest recorded sunlight-to-power efficiency in devices remains 13.0%, it is far lower than the theoretical efficiency of 32% [4]. This large gap is principally due to their low open-circuit voltage (V_oc_) and low fill factor (FF), which largely originate from the absorber layer’s quality [5,6,7]. Therefore, a top priority in the advancement of solar cells is obtaining a high-quality absorption layer. Extensive efforts have been devoted to this end, such as using the precise control of the ratios of Zn/Sn and Cu/(Zn+Sn) to boost the crystal growth and discard the secondary phases [8], using the substitution of extrinsic cations (i.e., Na, Cd, Ag, Mg, and others) to regulate the defects and crystal quality [9,10,11,12,13], and modification of the back interface (Mo/CZTSSe) to electrical contact/promote physical and prevent the formation of secondary phases [14,15]. Only the compact and uniform film surface contributes to the photoresponse of CZTSSe solar cells. This necessitates the removal of small grains and surface inhomogeneities to attain a higher power conversion efficiency (PCE).

To ameliorate the crystallinity of CZTSSe thin films, we successfully fabricated Cu_2_Mn_x_Zn_1−x_Sn(S,Se)_4_ (CMZTSSe) with various Mn amounts using the sol-gel method in our previous work [16]. However, thermal treatment was an indispensable step in the synthesis. In addition, annealing affects the composition of CZTSSe, rendering it a suitable method to regulate the composition of the films. An important composition parameter that requires restraint is the ratio of S/(S+Se) [8]. The bandgap of CZTSSe could be regulated from 1.0 eV (CZTSe) to 1.5 eV (CZTS) by altering the ratio of S/Se. The S/Se ratio has an influence on crystallization and purity; it has been proven that a higher S/Se ratio can enable the generation of defects and a secondary phase, which can damage a device’s properties [17,18]. In the process of annealing, S or Se are incorporated into the film and replace one another, allowing the film to have a different ratio of S/Se [19]. Adjusting the annealing temperature is a valid way to regulate the S/Se ratio of CZTSSe films. At present, the effects of a post-annealing treatment on the performances of the CZTSSe films have been reported in many works. For example, Zhuang et al. deposited CZTSSe absorber films with high Se/(Se+S) ratios by adjusting the selenization temperature [20]. They found that the CZTSSe films that were selenized at 460 °C had a layer of fine grains at the bottom of the CZTSSe absorber, and the average Voc of the CZTSSe solar cells increased from 284 mV to 371 mV. Lokhande et al. found that the composition, microstructure, and band gap of the Ge-doped CZTSSe films are highly sensitive to selenization conditions (temperature and time) [21]. The elemental (Ge and Sn) loss from the thin films increases with the increasing annealing temperature and time, and the Ge loss is more significant than Sn loss due to its low vapor pressure [21]. The results indicate that the post-annealing treatment is crucial for influencing the quality and properties of films. So far, the effects of the selenization temperature on the performances of Mn-doped CZTSSe films and solar cells have not been studied and reported in detail.

Hence, in this study, high-quality CMZTSSe absorber films were fabricated by synthesizing CMZTS precursor films using the sol-gel method, accompanied by a post-annealing treatment. Annealing was performed at temperatures from 500 °C to 560 °C under a Se/N_2_ environment. The results showed that dense and smooth CMZTSSe films could be acquired after the selenization-annealing process. The effects of the selenization temperature on the crystallinity and properties of the CMZTSSe films, as well as the photoelectric performance of the corresponding solar cell, were systematically studied.

## 2. Materials and Methods

### 2.1. Preparation of CMZTSSe Films

**Figure 1:**
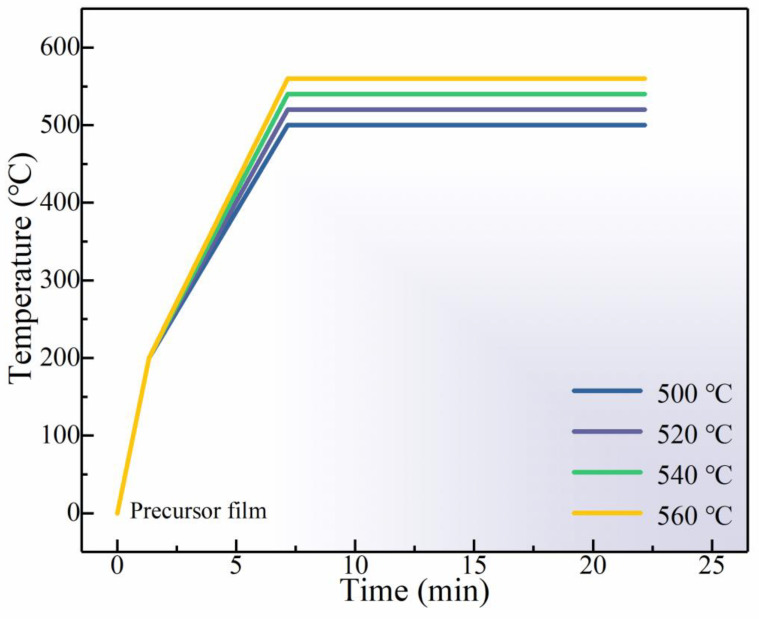
Detailed selenization process of CMZTS precursor films.

**Figure 2:**
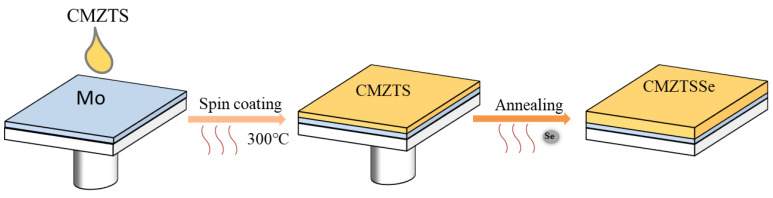
Schematic diagram of preparation process of CMZTSSe films.

Copper (II) acetate monohydrate (C_4_H_6_CuO_4_·H_2_O), zinc chloride (ZnCl_2_), manganese (II) chloride (MnCl_2_), tin chloride dihydrate (SnCl_2_·2H_2_O), dimethyl sulfoxide (DMSO), and thiourea (CH_4_N_2_S) were purchased from Aladdin Inc. C_4_H_6_CuO_4_·H_2_O (1.1979 g), ZnCl_2_ (0.5394 g), MnCl_2_ (0.0553 g), SnCl_2_·2H_2_O (0.8462 g), and CH_4_N_2_S (2.2836 g) were dissolved in 10 mL DMSO and mixed for 2 h to obtain a transparent yellow solution. Mo-coated soda lime glass (SLG) was used as substrate; the precursor solution was spin-coated at a speed of 3000 rpm for 29 s to prepare the CMZTS films, and then it was annealed on 300 °C hot plate for 3 min under a N_2_ environment. The processes of spin-coating and drying were duplicated 10 times. The CMZTS precursor film was then selenized in a graphite box under a N_2_ flow. The selenization procedure involved the following steps: increase the temperature to 200 °C in 80 s, heat to a certain selenization temperature (500 °C, 520 °C, 540 °C, and 560 °C) in 350 s, hold the temperature for 15 min, and enable natural cooling. The process is illustrated in Figure 1. The flowchart depicting the fabrication of the CMZTSSe films is shown in Figure 2.

### 2.2. Device Fabrication

SLG/Mo/CMZTSSe/CdS/ZnO/ITO/Ag solar cells were prepared by a conventional process. A 50 nm CdS was deposited on CMZTSSe layer by chemical bath deposition (CBD). Subsequently, using RF sputtering, an intrinsic ZnO layer with 50 nm thickness was deposited, and an Al-doped ZnO layer with 300 nm thickness was prepared. Finally, a Ag collection grid with 300 nm thickness was prepared on the top of the device by thermal evaporation.

### 2.3. Characterization

X-ray diffraction (XRD) was implemented using an X-ray diffractometer (Rigaku D/max ga X-ray diffractometer in Tokyo, Japan), wherein Cu Ka (λ = 0.15406 nm) was the radiation source. Raman spectra were recorded using a Renishaw system (Renishaw, London, UK) with a 514 nm excitation wavelength. Scanning electron microscopy (SEM) was performed using a Hitachi S-4800 (JEOL Ltd., Tokyo, Japan), which was provided with energy-dispersive X-ray spectroscopy (EDS) under 15 kV at various magnifications. The composition of the CMZTSSe was characterized by X-ray photoelectron spectroscopy (XPS) (Thermofisher, Waltham, MA, USA) with monochromated Al Kα radiation. An ultraviolet–visible–near-infrared (UV–vis–NIR) spectrophotometer (UV-3101PC, Tokyo, Japan) was used to characterize the optical properties of the CMZTSSe. A Hall-effect measurement system (Lake shore 7600 Hall, Irvine, CA, USA) was used to test the electrical performance. The current–voltage measurements under AM 1.5 G simulated sunlight illumination (Model 91160, Newport, Irvine, CA, USA) was performed to characterize the electrical properties of the solar cell. An external quantum efficiency (EQE) measurement system was applied to measure the spectral response of the device (QEX10, Newport, Irvine, CA, USA).

## 3. Results and Discussion

**Figure 3:**
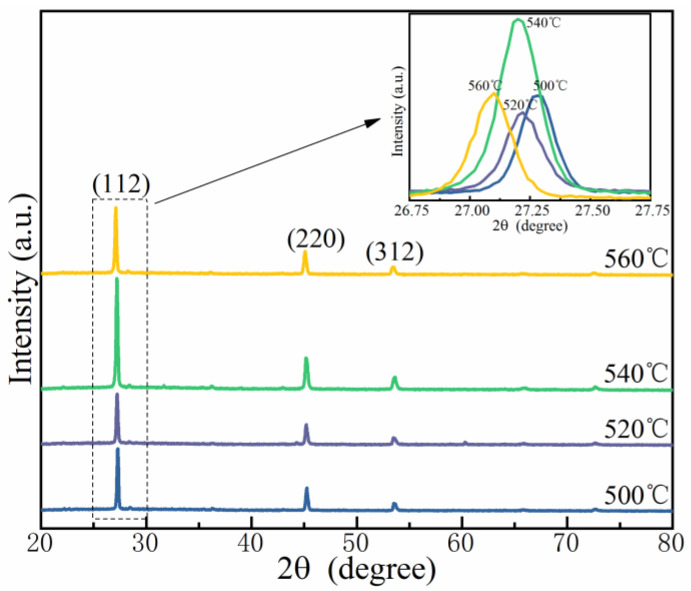
XRD patterns of the CMZTSSe films annealed at 500 °C, 520 °C, 540 °C, and 560 °C; Inset: Enlarged images of (112) peaks of all films.

Figure 3 displays the XRD profiles of the CMZTSSe films annealed at 500, 520, 540, and 560 °C. For all the films, three sharp peaks were observed in the XRD patterns, assigned to the diffractions of the (112), (220), and (312) crystal planes of Cu_2_ZnSnS_4_ (CZTS) with the kesterite phase, according to the International Center for Diffraction Data standards of JCPDS-26-0575 [22,23]. No diffraction peak of an impurity phase was found, indicating that the synthesized CMZTSSe films have a single-phase and kesterite structure at all selenization temperatures. As shown in the inset of Figure 3, the (112) diffraction peak shifts from 27.28° to 27.09° as the selenization temperature increases from 500 °C to 560 °C, manifesting an enlargement of the lattice owing to the considerable displacement of small S by a large Se atom while keeping the kesterite structure.

**Figure 4:**
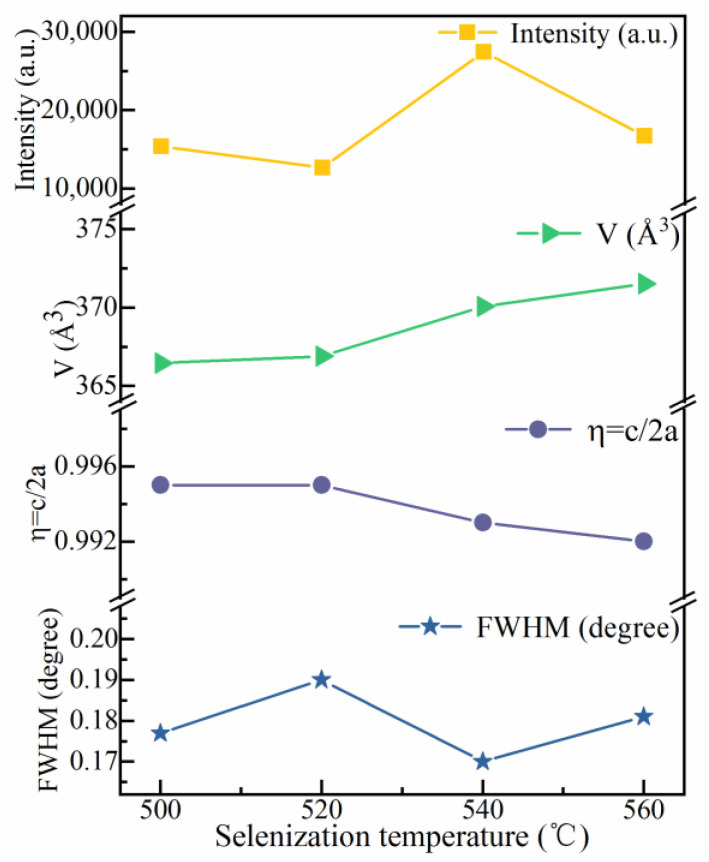
Curves of the FWHM and peak intensity of (112) peaks, unit cell volume (V), and lattice parameters (η = c/2a) for the CNZTSSe films as a function of selenization temperature.

Figure 4 shows the full width at half-maxima (FWHMs), lattice parameters (η = c/2a), unit cell volumes (V), and intensities of the (112) peak for the CMZTSSe films with different annealing temperatures, using the data obtained through the (112) peak in the XRD pattern. The FWHM is at its minimum when the selenization temperature is 540 °C. Obviously, for the intensity of the (112) peak, an opposite change trend is noticed simultaneously. When the temperature is increased up to 540 °C, the (112) peak intensity is maximal. This demonstrates that the film has the best crystallization quality at 540 °C. In Figure 4, the value of V increases with the increasing selenization temperature. This augmentation of the volume of the unit cell is due to the increment in the content of Se in the films. With the increasing selenization temperature, more S atoms are replaced by Se atoms with a larger radius, which will be proven later in the EDS results. The first calculation principles have demonstrated that η has distinct symmetries, i.e., η < 1 represents a kesterite structure, while η > 1 represents a stannite structure [24,25]. It was found that for all the CMZTSSe films, the η values were less than 1. Thus, it was confirmed that all the CMZTSSe films maintained the kesterite structure.

**Figure 5:**
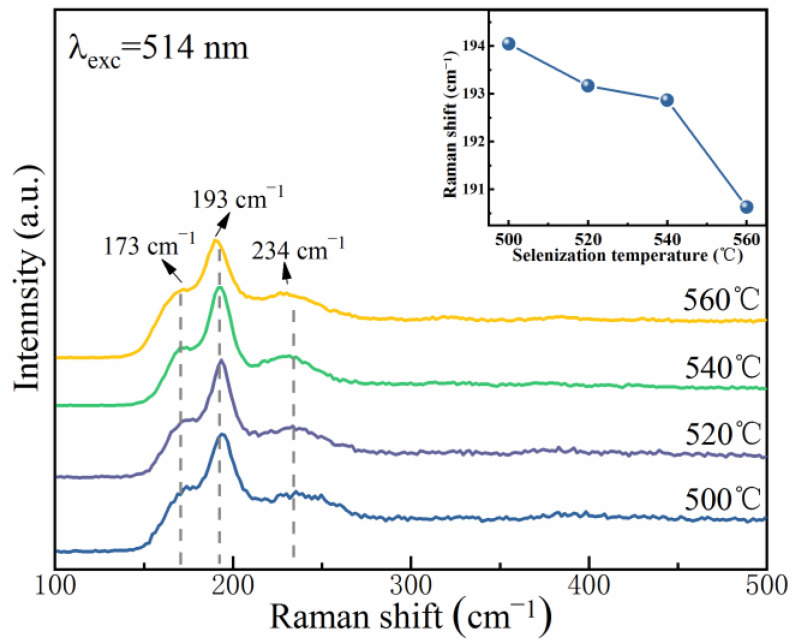
Raman spectra of CMZTSSe films annealed at various selenization temperatures; Inset: Variation curve of main Raman vibration mode A(1) with selenization temperature.

The XRD peaks of the CZTS(Se) films with a kesterite structure are extremely similar to those of cubic ZnS(Se) and ternary Cu_2_SnS(Se)_3_, making it very hard to identify the secondary phases using XRD [26]. Raman spectroscopy was used as a supportive method to identify secondary phases as it is sensitive to the vibrations of lattice atoms. Figure 5 exhibits the Raman spectra of the CMZTSSe films annealed at 500, 520, 540, and 560 °C. When the selenization temperature reaches 500 °C, Raman peaks are detected at 174.7, 194.0, and 235.7 cm^−1^, which match the Raman characteristic vibrations of the A2, A1, and B modes of the CZTSSe phase [27]. The Raman peaks of other probable secondary phases were not measured for all CMZTSSe films. These results also prove the formation of single-phase CMZTSSe films. At a selenization temperature of 520 °C, the peaks slightly shift to lower wavenumbers at 173.3, 193.1, and 234.5 cm^−1^. When the selenization temperature reaches 540 °C, the peaks shift toward 172.2, 192.9, and 231.4 cm^−1^. As the selenization temperature further rises to 560 °C, the peaks shift to lower wavenumbers of 170.3, 190.6, and 229.4 cm^−1^. Therefore, as the selenization temperature increased from 500 °C to 560 °C, all the Raman peaks moved to lower wavenumbers. The inset of Figure 5 tracks the peak positions of the A1 mode and shows that with the increasing selenization temperature, the peak of the A1 mode shifts to lower wavenumbers. This may be because of the substitution of some bigger Se atoms for S atoms, leading to an augmentation of the lattice constant, which is in accordance with the XRD results.

**Figure 6:**
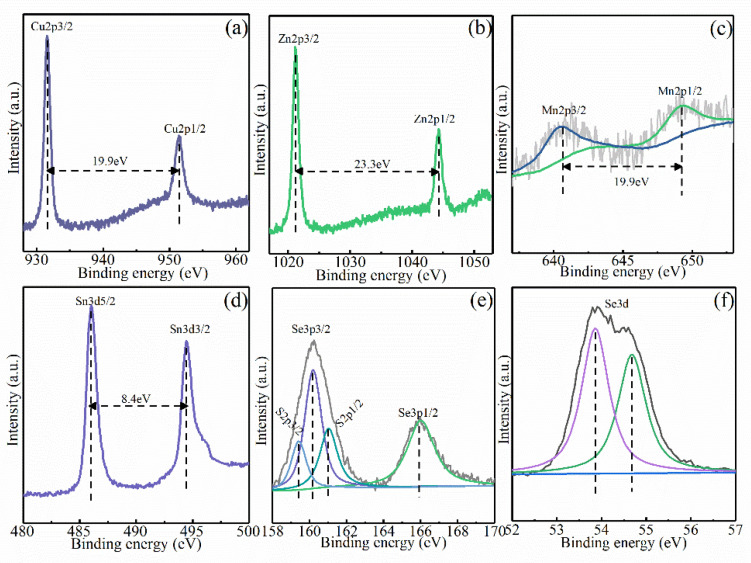
XPS spectra of CMZTSSe films annealed at 540 °C: (**a**) Cu 2p, (**b**) Zn 2p, (**c**) Mn 2p, (**d**) Sn 3d, (**e**) S2p, and (**f**) Se 3d.

**Figure 7:**
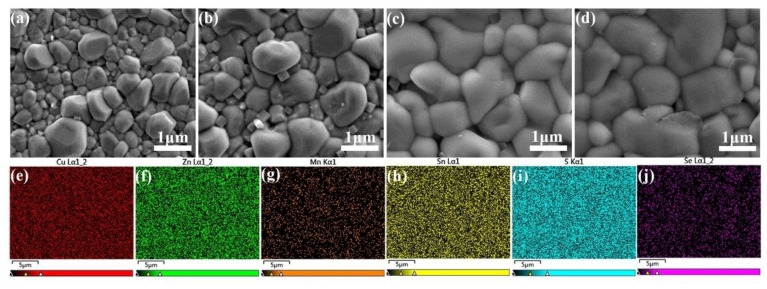
SEM surface images of the CMZTSSe films annealed at 500 °C (**a**), 520 °C (**b**), 540 °C (**c**), and 560 °C (**d**); EDS mapping images for Cu, Zn, Mn, Sn, S, and Se elements in the CMZTSSe film annealed at 540 °C (**e**–**j**).

The chemical valence state of the constituent elements in the CMZTSSe films annealed at 540 °C was determined by XPS. Figure 6a–f shows the XPS profiles of elements including Zn, Cu, Mn, Sn, S, and Se, as well as their fitted curves for the CMZTSSe films annealed at 540 °C. The XPS profile of the Cu 2p is displayed in Figure 6a. There are two XPS peaks situated at 931.6 and 951.5 eV, corresponding to Cu 2p3/2 and Cu 2p1/2. The interval of energy is 19.9 eV, suggesting that Cu^+^ lies in the CMZTSSe film [28]. Figure 6b presents the Zn 2p profile, where the peak of Zn 2p3/2 is located at 1021.4 eV, and the peak of Zn 2p1/2 is located at 1044.5 eV. The peak-spacing value is 23.1 eV, implying that Zn occurs in the +2 valence state [29,30]. In Figure 7c, the XPS profile of Mn 2p presents two peaks at 640.6 eV and 649.2 eV, corresponding to Mn 2p_3/2_ and Mn 2p_1/2_ with an energy gap of 8.6 eV, coinciding with the binding energy value of Mn^2+^ [31]. Figure 7d shows the Sn 3d XPS profile. The two XPS peaks situated at 486.0 eV and 494.5 eV are ascribed to Sn 3d_5/2_ and Sn 3d_3/2_, and the energy gap is 8.5 eV, which implies that the valence state of Sn is +4 Since the S 2p core level and Se 3p core level nearly overlap, in Figure 7e, Gaussian fitting was used to fit the profile into four peaks situated at 159.4, 160.2, 161.1, and 165.9 eV, attributed to Se 2p_3/2_, S 2p_3/2_, S 2p_1/2_, and Se 3p_1/2_. The S 2p3/2 (160.2 eV) and S 2p1/2 (161.1 eV) peaks are in the representative reference standard value scope (160–164 eV), implying that S lies in S^2−^ [32]. In Figure 7f, the XPS profile of Se 3d was Gaussian-fitted into two peaks at 53.9 and 54.7 eV, corresponding to Se 3d_3/2_ and Se 3d_1/2_. The peak-spacing value is the same as that of standard Se^2−^ [33]. The XPS results indicate that the component elements (Cu, Zn, Mn, Sn, S, and Se) are in the form of Cu^1+^, Zn^2+^, Mn^2+^, Sn^4+^, S^2−^, and Se^2−^, respectively, in the CMZTSSe films.

Table 1 lists the atomic percentages of the constituent elements in the CMZTSSe films annealed at 500, 520, 540, and 560 °C. The EDS results show that with the increasing selenization temperature, the atomic percentage of Se gradually increases, whereas that of S gradually decreases. This is mainly due to the greater number of Se atoms replacing the S atoms with the increasing selenization temperature. When the selenization temperature was raised to 520 °C and 540 °C, the ratio of Mn/(Zn+Mn) was slightly lower. The reduction in this ratio occurs to a lesser extent for the CMZTSSe film annealed at 540 °C; hence, it retains the compositional and microstructural quality of the thin film. As the selenization temperature increases to 560 °C, the elemental loss is significant. Zn and Mn are lost in excess, which affects the composition and microstructure of the films. This loss is due to the evaporation of Mn and Zn in the form of sulfide/selenide [14,34], e.g., Mn(Se,S), Mn(Se,S)_2_, Zn(Se,S), and Zn(Se,S)_2_, with the increasing selenization temperature. Thus, 540 °C can be considered the optimal selenization temperature.

An appropriate selenization temperature is significant in order to gain large crystal particles and few voids in the CMZTSSe films. The SEM images of the CMZTSSe films annealed at 500, 520, 540, and 560 °C are presented in Figure 7a–d, respectively. Figure 7a,b show the SEM images of the CMZTSSe films annealed at 500 and 520 °C, respectively. In these figures, the surface of the CMZTSSe film is comparatively coarse, with nonuniform small grains sized 200–600 nm. When the temperature is raised to 540 °C, there is an obvious change in the surface morphology of the grains (Figure 7c), whereby the grain size increases markedly to 1.0–1.5 μm and the CMZTSSe film presents a very smooth and dense state, implying enhanced crystallization due to the increase in the selenization temperature. Figure 7d displays that when increasing the temperature to 560 °C, the crystal sizes become somewhat bigger. However, the surface is in a non-dense state, and there are some voids and holes. Thus, regarding the effect of the crystallinity of the CMZTSSe on the PCE, the best selenization temperature is 540 °C in the present work. Figure 7e–j show the EDS elemental-mapping images of the CMZTSSe film annealed at 540 °C. The EDS-mapping images reveal a uniform distribution of Cu, Zn, Mn, Sn, S, and Se throughout the CMZTSSe film.

**Figure 8:**
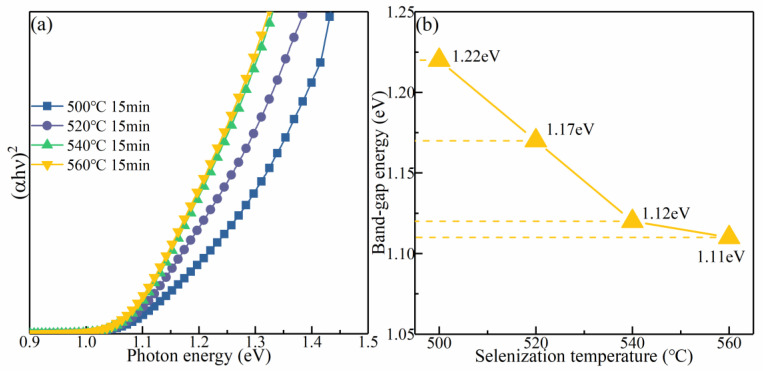
(**a**) The plot of (αhυ)^2^ against hυ for CMZTSSe films annealed at 500 °C 520 °C, 540 °C, and 560 °C; (**b**) The variation in bandgap for the CMZTSSe films as a function of the selenization temperature.

To survey the influence of the selenization temperature on the optical bandgap (Eg), the absorption spectra of the samples were acquired by a UV–vis–NIR spectrophotometer. Figure 8a presents the change in (αhν)^2^ for the CMZTSSe films annealed at distinct selenization temperatures (500, 520, 540, and 560 °C) with the photon energy (hν). For the direct bandgap semiconductor, the correlation between the absorption coefficient (α) and Eg was determined by the formula:

α (hν) = B[(hν − Eg)^1/2^/hν] (1)

where B is a parameter related to the states of the band tail [35]. According to the formula (1) and the data in Figure 8a, the Eg values of the CMZTSSe films annealed at different temperatures (500, 520, 540, and 560 °C) are 1.22, 1.17, 1.12, and 1.11 eV, respectively. Figure 8b shows that the Eg decreases with the increasing selenization temperature. By collectively considering the XRD and EDS results, this decrease in Eg may be due to the displacement of S by Se, which leads to changes in the lattice parameter and electronegativity after the atomic structure is alloyed and modified. In addition, it is known that the Eg of CZTS is larger than that of CZTSe [36]. Thus, the Eg of CMZTSSe approximates the Eg of CZTSe when the content of Se increases. Furthermore, based on first-principles calculations, with the increasing selenization temperature, more Se atoms replace S atoms, and the interaction of the orbit between the conduction band minimum (CBM) and the valence band maximum (VBM) is degraded, resulting in a decrease in the Eg.

The electrical properties of the CMZTSSe films annealed at various temperatures (500, 520, 540, and 560 °C) were measured using a Hall-effect apparatus, as displayed in Table 2. The CMZTSSe films all exhibit p-type electrical conductivity. As the selenization temperature increases from 500 to 540 °C, the carrier concentration first increases from 2.95 × 10^15^ to 6.97 × 10^16^ cm^−3^, but then decreases to 7.53 × 10^15^ cm^−3^ at 560 °C. The mobility decreases from 4.35 cm^−2^V^−1^s^−1^ at 500 °C to 1.93 cm^−2^V^−1^s^−1^ at 540 °C, but increases to 3.90 cm^−2^V^−1^s^−1^ at 560 °C. Meanwhile, the resistivity initially decreases from 4.89×10^2^ Ω·cm at 500 °C to 3.74 × 10^1^ Ω·cm at 540 °C, then increases to 2.19 × 10^2^ Ω·cm at 560 °C. This signifies that the CMZTSSe film has the best electrical properties at 540 °C. At this temperature, the carrier concentration reached its maximum value, and the resistivity attained its minimum value owing to the enhancement in the crystallinity of the film, and the decrease in the recombination of the carrier at grain boundaries, which remarkably increases the number of the carrier concentration [37]. Nevertheless, as the carrier concentration increases, the scattering of the carrier enhances, leading to a reduction in mobility [38].

**Figure 9:**
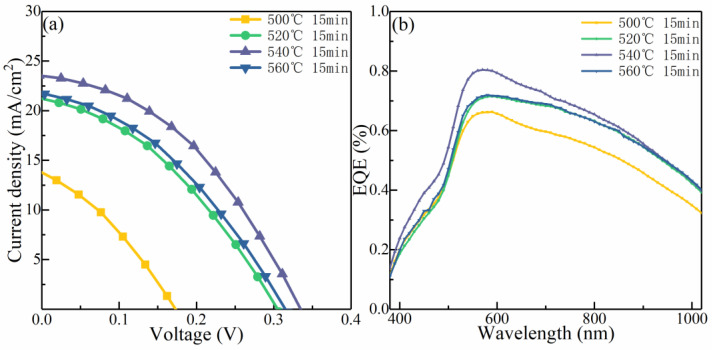
(**a**) Current–voltage characteristics of the CMZTSSe-based solar cells synthesized using the CMZTSSe absorber layer annealed at the temperatures of 500 °C, 520 °C, 540 °C, and 560 °C. (**b**) EQE spectra of the corresponding CMZTSSe-based solar cells.

Figure 9a shows the current–voltage (J–V) characteristic profiles of the CMZTSSe devices synthesized at various selenization temperatures (500, 520, 540, and 560 °C). The corresponding device performance parameters are presented in Table 3. The PCE increases from 0.70% to 3.08% as the selenization temperature increases from 500 °C to 540 °C, and then decreases to 2.24% with a further increase in the temperature to 560 °C. The variations in V_oc_, J_sc_, and the FF with the PCE are similar, as shown in Table 3. When increasing the selenization temperature from 500 °C to 540 °C, V_oc_, J_sc_, and FF increase significantly, and then decrease from 540 °C to 560 °C. These variations in the device performance parameters may result from the change in the crystallinity of the absorber layer and the alteration in the optical bandgap induced by annealing at different selenization temperatures. Figure 9b shows the EQE spectra of the devices utilizing CMZTSSe as the absorber layers synthesized at selenization temperatures of 500, 520, 540, and 560 °C. The EQE enhances distinctly in the 350–1100 nm wavelength range as the temperature increases from 500 to 540 °C. The enhancement in the EQE is principally due to the optimization of the absorber layer films, which allows for a growing number of photons to be incorporated [39,40]. Moreover, the enhancement in the EQE implies that the collection of charge is improved in the region space of the charge, and the recombination of the carrier is decreased [41]. However, the spectral response of the EQE decreases at 560 °C. This may be ascribed to two reasons. On the one hand, the CMZTSSe phase may decompose under the condition of a higher selenization temperature. On the other hand, a poor p–n junction may form due to the increasing deterioration of the film quality [42].

## 4. Conclusions

In conclusion, pure-phase CMZTSSe films were acquired by the simple sol-gel method and post-annealed CMZTS precursor films. The effect of the selenization temperature on the device performance was surveyed by analyzing the change in the crystallization quality and the photoelectric properties of the CMZTSSe films. The results indicated that the Eg of CZTSSe could be changed in the range from 1.22 eV to 1.11 eV with the increase in the selenization temperature from 500 to 560 °C. All the CMZTSSe films displayed p-type electrical conductivity, and by changing the selenization temperature, films with optimal conduction properties could be achieved. When the selenization temperature was 540 °C, the CMZTSSe films acquired the preferred crystal quality as well as electrical and optical properties and acted as the absorber layer to fabricate a solar cell. Finally, the best PCE of 3.08% was achieved with a Voc of 334 mV, a Jsc of 23.46 mA/cm^2^, and an FF of 39.30% when the selenization temperature was 540 °C.

## Figures and Tables

**Table 1:** EDS results of the CMZTSSe films annealed at different temperatures from 520 to 560 °C.

Temperature (°C)	Cu (at%)	Mn (at%)	Zn (at%)	Sn (at%)	S (at%)	Se (at%)	Se/(S + Se)	Cu/(Zn + Mn + Sn)	Mn/(Mn + Zn)
500	23.87	1.23	11.35	14.46	2.78	46.32	0.94	0.88	0.08
520	23.44	0.96	11.43	14.37	2.73	46.87	0.95	0.87	0.07
540	23.57	1.11	11.40	14.47	2.57	46.89	0.95	0.87	0.07
560	23.76	1.01	10.96	15.86	2.07	47.34	0.96	0.85	0.06

**Table 2:** Electrical properties of the CMZTSSe films annealed at different temperatures from 520 to 560 °C.

Temperature (°C)	p (Ω·cm)	n (cm^−3^)	μ (cm^−2^V^−1^s^−1^)	Conduction Type
500	4.89 × 10^2^	2.95 × 10^15^	4.35	p
520	2.89 × 10^2^	5.58 × 10^15^	3.99	p
540	3.74 × 10^1^	6.97 × 10^16^	1.93	p
560	2.19 × 10^2^	7.53 × 10^15^	3.90	p

**Table 3:** The performance parameters of the CMZTSSe film solar cells.

Temperature (°C)	Active area (cm^2^)	V_OC_ (mV)	J_SC_ (mA/cm^2^)	FF (%)	PCE (%)
500	0.19 cm^2^	173	13.82	29.20	0.70
520	0.19 cm^2^	304	21.11	31.62	2.03
540	0.19 cm^2^	334	23.46	39.30	3.08
560	0.19 cm^2^	315	21.67	33.40	2.24
